# Encounter rates prime interactions between microorganisms

**DOI:** 10.1098/rsfs.2022.0059

**Published:** 2023-02-10

**Authors:** Jonasz Słomka, Uria Alcolombri, Francesco Carrara, Riccardo Foffi, François J. Peaudecerf, Matti Zbinden, Roman Stocker

**Affiliations:** Department of Civil, Environmental and Geomatic Engineering, Institute of Environmental Engineering, ETH Zurich, Zurich, Switzerland

**Keywords:** encounter rates, microbial interactions, encounter kernels

## Abstract

Properties of microbial communities emerge from the interactions between microorganisms and between microorganisms and their environment. At the scale of the organisms, microbial interactions are multi-step processes that are initiated by cell–cell or cell–resource encounters. Quantification and rational design of microbial interactions thus require quantification of encounter rates. Encounter rates can often be quantified through encounter kernels—mathematical formulae that capture the dependence of encounter rates on cell phenotypes, such as cell size, shape, density or motility, and environmental conditions, such as turbulence intensity or viscosity. While encounter kernels have been studied for over a century, they are often not sufficiently considered in descriptions of microbial populations. Furthermore, formulae for kernels are known only in a small number of canonical encounter scenarios. Yet, encounter kernels can guide experimental efforts to control microbial interactions by elucidating how encounter rates depend on key phenotypic and environmental variables. Encounter kernels also provide physically grounded estimates for parameters that are used in ecological models of microbial populations. We illustrate this encounter-oriented perspective on microbial interactions by reviewing traditional and recently identified kernels describing encounters between microorganisms and between microorganisms and resources in aquatic systems.

## 1. Introduction

Microbial communities impact human health [1], global biogeochemical cycles [2,3] and plant growth [4]. Properties of microbial communities, such as resilience, coexistence and self-organization, emerge from the interactions between their members and between members and the environment [5,6]. Studying this emergence is challenging due to the complex and dynamic nature of the interactions [5]. To tackle this complexity, the merger of experimental approaches and mathematical modelling is key [5,6].

**Figure 1.**
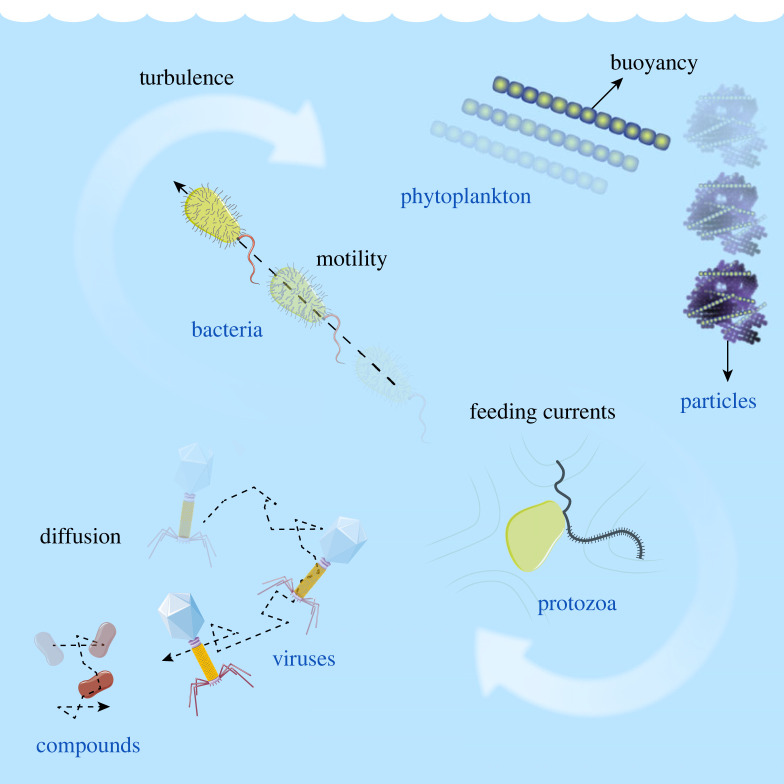
One litre of seawater contains a myriad of particles of organic matter, phytoplankton and protozoa cells, bacteria, viruses and dissolved chemical compounds. These different objects (drawn not to scale) can encounter one another, for example, due to fluid mixing, density mismatch, motility and diffusion.

**Figure 2.**
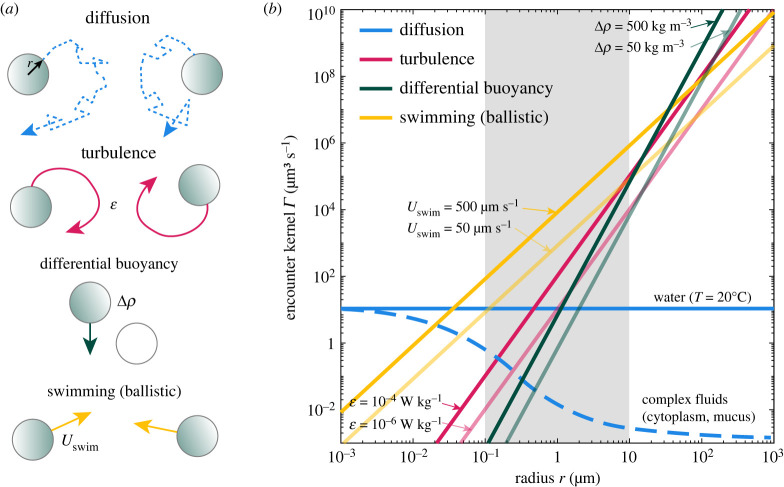
Even in the simplest encounter models that represent objects (cells, particles, compounds) as identical spheres (*a*), the encounter rates strongly depend on encounter mechanisms, cell phenotypes and environmental conditions (*b*). The confluence of encounter rates at micrometre scale (shaded area, note the constant blue curve), analogous to the confluence of energy scales at which molecular machines operate [28], illustrates the challenge of analysing microbial interactions and could contribute to microbial diversity.

**Figure 3.**
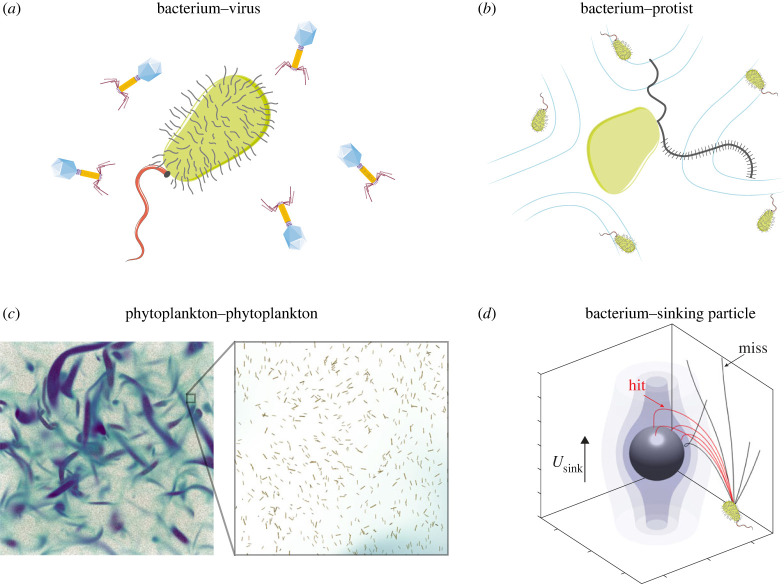
Encounters of cells with resources and other microorganisms control the timescales of viral infections of bacteria, predation by protists on bacteria, marine snow formation by coagulation of phytoplankton cells following a phytoplankton bloom, and bacterial colonization of sinking marine snow, among many other microbial interactions. (*a*) Bacterium–virus encounters are driven by viral diffusion and bacterial swimming. (*b*) Bacterium–protist encounters result from the swimming of protists and the feeding currents created by beating of their flagella. (*c*) Phytoplankton–phytoplankton encounters are driven by buoyancy and turbulent mixing. The colour code shows intense vorticity structures visualizing locally swirling regions in the turbulent flow. (*d*) Bacterium–particle encounters result from the sinking of the particle, bacterial swimming and flow-induced reorientation of the bacterial swimming trajectory. (*c*) Reproduced from [29] (published under CC BY-NC-ND 4.0 license). (*d*) Adapted from [9] (published under CC BY 4.0 license).

At the scale of the organisms, cell–cell and cell–resource encounters are essential first steps of microbial interactions [7]. Microbial degradation of particles of organic matter [8–10] or dispersed oil droplets [11] is initiated by bacteria–particle or bacteria–droplet encounters. Aggregation of gut bacterial populations [12,13] or colony formation by phytoplankton in the ocean [14,15] requires cell–cell encounters. Horizontal gene transfer relies on bacteria–bacteria, bacteria–virus or bacteria–DNA encounters [16]. Predation by protists on bacteria is an essential element in aquatic microbial food webs controlled by flows generated by protists to increase encounters with bacteria [17,18]. Marine snow formation by dead or senescent phytoplankton cells, a key component of the ocean biological pump, is driven by cell encounters [19–21]. Nutrient uptake [22] and exchange of metabolites [23] require diffusive encounters between cells and molecules. Additional examples include mating [24], fertilization [25] and finding symbiotic partners [26]. Quantifying encounter rates is thus an important step in quantifying microbial interactions [27], and, we argue, offers an important bridge between experimental investigations and mathematical modelling. To illustrate this encounter-oriented perspective, we here focus on encounters in aquatic environments (figure 1). We highlight the dependence of encounter rates on encounter mechanisms, microbial phenotypes and environmental parameters, and show that, for representative values of these parameters, the encounter rates for different encounter mechanisms approximately converge at the micrometre scale (figure 2). We discuss how this confluence of encounter rates could be a driver of microbial diversity. We then describe several examples of microbial interactions where estimating encounter rates enables one to make predictions of the timescales characterizing the interactions, which is key to testing hypotheses on mechanisms at play (figure 3). Throughout, we discuss recent progress and open problems, highlighting the potential and prolificacy of the encounter-centric approach to the study of microbial interactions.

## 2. Quantification of encounter rates

Water in the oceans’ euphotic zone contains a plethora of microorganisms and dissolved or particulate organic matter, which continuously encounter one another due to diffusion, advection by fluid flow, stirring by turbulence, buoyancy and motility (figure 1). In 1 l of seawater, there are tens of particles of organic matter larger than 100 μm [30,31], a million phytoplankton cells larger than 5 μm [32] and protozoa [33,34], a billion bacteria [35,36], 10 billion viruses [35,36] and an often heterogeneous continuum of dissolved chemical compounds.

These different objects (chemicals, organisms or particles), when distributed homogeneously, encounter one another at a rate that is predicted by the following equation:

2.1 $$\text{encounter rate per unit volume}=\Gamma c_{A}c_{B},$$

where *c*_A_ and *c*_B_ are the concentrations of the two objects encountering each other. For example, A could be a species of bacteria and B the viruses that infect it. Equation (2.1) captures the intuitive idea that the encounter rate is proportional to concentrations (i.e. doubling the concentration of each object results in a fourfold increase in encounter rates). The factor Γ is called the encounter kernel. The encounter kernel has units of volume per time and represents the volume that a pair of encountering objects sweep relative to each other per unit time. Our focus here is on how the encounter kernel depends on the mechanisms that bring the two objects together, the characteristics of the objects (e.g. organism phenotypes) and the environmental conditions. Once the encounter kernel is known, equation (2.1) enables one to predict the encounter rate for given concentrations of the two objects, *c*_A_ and *c*_B_. Equation (2.1) has been widely applied to study rates of chemical reactions (compound–compound encounters) [37], bacterium–virus adsorption [38], nutrient uptake (cell–compound encounters) [22], marine snow formation (cell–cell encounters) [19] and predator–prey dynamics [39]. For clarity, we stress that equation (2.1) quantifies the encounter stage of an otherwise multi-step interaction process. For example, equation (2.1) can predict encounter rates between phytoplankton cells but it does not determine whether or not cells stick after an encounter, and hence aggregate, or it can predict diffusive uptake of signalling molecules (e.g. in quorum sensing) but it does not capture the regulatory response of cells.

Before moving to specific encounter mechanisms, we note that equation (2.1) represents the encounter rate between any object A with any object B. For example, equation (2.1) predicts that an experimentalist counting all encounters between bacteria (of concentration *c*_bac_) and phytoplankton (of concentration *c*_phy_) during time *T* in an observation domain of volume *V* should obtain, on average, $\Gamma c_{\mathrm{bac}}c_{\mathrm{phy}}VT$ encounters. An important variant of equation (2.1) concerns the perspective of an individual object: any given individual bacterium encounters phytoplankton cells with rate $\Gamma c_{\mathrm{phy}}$ and vice versa, any given phytoplankton cell encounters bacteria with rate $\Gamma c_{\mathrm{bac}}$. We finally note that equation (2.1) is deterministic and has no dependence on space: it is thus limited to well-mixed, spatially homogeneous systems where each object is present at high copy number—we discuss generalizations to stochastic and heterogeneous systems later.

## 3. Encounter mechanisms in aquatic systems

Each mechanism that generates encounters, such as diffusion, fluid flow, buoyancy and motility (figure 1), is characterized by a different encounter kernel. As a simplification, we now make (and later will relax) the spherical cell assumption, namely we model the objects A and B as being spherical and all having the same radius within each species, *r*_A_ and *r*_B_, respectively. We can then obtain mathematical expressions for the encounter kernels linked to different mechanisms.

Diffusive encounters are then characterized by the kernel [40,41]

3.1 $$\Gamma_{\mathrm{diff}}=4\pi (D_{A}+D_{B})(r_{A}+r_{B}),$$

where *D*_A_ and *D*_B_ are the thermal diffusion coefficients of the objects. From the Stokes–Einstein relation, *D*_A_ = *k*_B_*T*/(6*πμr*_A_), where *k*_B_ is the Boltzmann constant, *T* is the temperature and *μ* is the dynamic viscosity of the fluid (typically water, in which case *μ* = 1 mPa s at *T* = 20°C). Equation (3.1) is based on the assumption that objects act as perfect absorbers and it neglects the initial transient encounters due to the buildup of boundary layer [39–41]. The impact of imperfect absorption has been studied in great detail in the classic paper by Berg & Purcell [38], where they showed that only a small fraction of the surface of an object needs to be absorbing in order achieve a nearly optimal performance.

Encounters in turbulence are characterized by [42]

3.2 $$\Gamma_{\mathrm{turb}}=1.3{\left(r_{A}+r_{B}\right)}^{3}\sqrt{\frac{\epsilon }{\nu }}\;,$$

where ϵ is the kinetic energy dissipation rate characterizing turbulence intensity and *ν* is the kinematic viscosity of the fluid (*ν* = *μ*/*ρ*, where *ρ* is the fluid density; *ν* = 1 mm^2^ s^−1^ for water at *T* = 20°C). Equation (3.2) is valid for objects smaller than the so-called Kolmogorov scale, which in the ocean is typically larger than 1 mm [43].

The kernel for cells moving vertically along the water column, for example, due to buoyancy, is [44]

3.3 $$\Gamma_{\mathrm{buoy}}=\pi {\left(r_{A}+r_{B}\right)}^{2}|U_{A}(r_{A})-U_{B}(r_{B})|,$$

where *U*(*r*) is the vertical velocity, which in general differs between the objects A and B, causing differential settling. The vertical velocity can be positive (rising) or negative (sinking), and typically depends on the radius *r*, density offset Δ*ρ* with respect to the surrounding fluid, gravity *g* and dynamic fluid viscosity *μ*, as described by Stokes’ law: *U*(*r*) = 2Δ*ρgr*^2^/(9*μ*) (note that the sinking of marine particles has been found to obey more closely empirical relations than Stokes’ law [45]).

For motile cells, all moving in straight lines at the same speed *U*_swim_ but in random directions, the kernel reads [44]

3.4 $$\Gamma_{\mathrm{mot}}=\frac{4}{3}\pi {\left(r_{A}+r_{B}\right)}^{2}U_{\mathrm{swim}}.$$

When the speeds are unequal between A and B, the kernel becomes $\Gamma_{\mathrm{mot}}=\pi {\left(r_{A}+r_{B}\right)}^{2}(3U_{A}^{2}+U_{B}^{2})/(3U_{A})$ with the condition *U*_A_ ≥ *U*_B_ [46,47]. When the speeds are randomly distributed, rather than being constant, the kernel is known in the case of Maxwell’s distribution (i.e. each velocity component is normally distributed with zero mean). In that case, with distribution means ${\bar{U}}_{A}$ and ${\bar{U}}_{B}$, the kernel is $\Gamma_{\mathrm{mot}}=\pi {\left(r_{A}+r_{B}\right)}^{2}\sqrt{{\bar{U}}_{A}^{2}+{\bar{U}}_{B}^{2}}$ [44,47].

For motile cells, we note that equation (3.4) and its variants assume straight-line, ballistic motion. When cells reorient after travelling for some typical distance *λ*_A_ and *λ*_B_, equation (3.4) holds as long as *λ*_A,B_ ≫ *r*_A,B_ [39]. That is, the encounter process must appear ballistic from the perspective of encountering cells (run length greater than cell size). When this condition is violated, the encounters become increasingly diffusive [48–50] and the kernel is described by equation (3.1), with thermal diffusion coefficients replaced by the effective diffusion coefficients associated with the cells’ active motion [51,52].

Not only are kernels specific to each encounter mechanism, but they also strongly depend on phenotypes of cells or characteristics of particles, and environmental conditions (figure 2). To illustrate these dependencies, we set *r*_A_ = *r*_B_ = *r* in equations (3.1)–(3.4) to focus, in the next four paragraphs, on the simplest case where all objects (cells or particles) have the same size.

Diffusive encounters between equal-sized spheres in water do not depend on cell size, as shown by combining equation (3.1) with the Stokes–Einstein relation, which yields $\Gamma_{\mathrm{diff}}=8k_{B}T/(3\mu )$. Since encounter rates for all other encounter mechanisms decrease as the object size decreases, diffusive encounters rule the submicrometre world, and are still important for micrometre-sized objects (blue line in figure 2*b*). Interestingly, diffusion in complex fluids, such as cytoplasm or human mucus, is scale dependent [53,54]. Viscosity in such environments increases rapidly with the size of the diffusing object, which can suppress diffusive encounters for larger objects, making them relevant only at the nanometre scale (broken blue line in figure 2*b*). Suppression of diffusion in mucus is a first-line protection mechanism against foreign pathogens [53], in bacterial extracellular polymeric substances it can protect bacteria from external deleterious compounds [55] and has been observed outside phytoplankton cells [56].

Encounters in turbulence between equal-sized spheres depend strongly on size, as seen from the cubic scaling $\Gamma_{\mathrm{turb}}=10.4r^{3}\sqrt{\epsilon /\nu }$ (pink line in figure 2*b*). At intermediate ($\epsilon =10^{-6}\;W\;{\mathrm{kg}}^{-1}$) and strong ($\epsilon =10^{-4}\;W\;{\mathrm{kg}}^{-1}$) turbulence intensities [43], turbulence dominates diffusion for encounters between objects with size above 1 μm and 0.5 μm, respectively.

Equal-size spherical objects with the same density offset with respect to the surrounding fluid do not encounter one another because of differential settling (i.e. $\Gamma_{\mathrm{buoy}}=0$), because they all settle or rise at the same speed. Instead, we consider an example where the cells are split into two equal subpopulations: neutrally buoyant (stationary) cells and cells with density offset Δ*ρ*. This scenario is relevant, for example, for phytoplankton cells that can actively control their buoyancy [14] and whose subpopulations may thus have different buoyancy levels. Using Stokes’ law and including a factor of 1/2 to account for the two half-populations, the kernel becomes $\Gamma_{\mathrm{buoy}}=4\pi r^{4}\Delta \rho g/(9\mu )$ (green line in figure 2*b*). Due to the strong dependence on the size of an object (quartic scaling), whenever differential buoyancy is established, it may be the dominant encounter mechanism for large (greater than 100 μm) objects.

Finally, swimming in straight lines generates encounters that scale with the square of object size, $\Gamma_{\mathrm{mot}}=16\pi r^{2}U_{\mathrm{swim}}/3$ (yellow line in figure 2*b*), and may be the dominant encounter mechanism at the micrometre scale, because it induces large relative velocities between encountering objects. For example, at the scale of a micrometre, unrealistically strong turbulence or density offsets are needed to produce relative speed of the order of tens of micrometres per second. We note that the swimming speeds of microorganisms can vary by orders of magnitude, and depend on a range of factors including cell size, shape, number of flagella (or cilia), actuation frequencies, fluid temperature, and viscosity and metabolic state of the cell, among others [57–60]. In figure 2, we captured some of this variability by focusing on two values, which can be broadly seen as characteristic of bacteria (*U*_swim_ = 50 μm s^−1^) and dinoflagellates (*U*_swim_ = 500 μm s^−1^) [60] (even though each group of organisms itself exhibits large variations).

## 4. Confluence of encounter mechanisms

Figure 2 shows an interesting feature of encounter rates: a convergence of the magnitude of multiple encounter mechanisms at the size of an object in the range of 1 μm (shaded area). This confluence of encounter rates bears conceptual analogy with the confluence of the magnitude of multiple interaction energies (thermal, chemical, electrostatic, bending) at the size of an object in the nanometre range [28]. For that case, since molecular machines operate at the nanometre scale, it has been proposed that this confluence may be responsible for the rich functionality of molecular machines: the confluence enables conformational changes, dissolution of bonds, transport of charges, which in turn are the basis of macromolecular functions such as DNA reading and copying or action of molecular motors [28].

Following this analogy, we propose that it might be plausible that the large microbial diversity observed in nature [61,62] could be in part driven by the confluence of encounter mechanisms at the scale of individual microorganisms (approx. 1 μm). This specifically would mean that different microorganisms can achieve the same function (an encounter with a given entity, e.g. a resource or another microorganism) through different encounter mechanisms. This would in turn require different adaptations (e.g. motile versus non-motile cells; smaller versus larger cells) that contribute to microbial diversity. Species diversity and coexistence are known to be promoted by environmental fluctuations [63], competition [64], predation [65] and coevolution [66] (see also discussion in [7,67]). The confluence of encounter mechanisms at the microbial scale adds a possible additional driver of diversity: since microbial interactions can be mediated by many different encounter mechanisms, rather than being dominated by a single one, this multiplicity may facilitate diverse life strategies of microbes, eventually promoting diversity. At a coarse level, this is already visible in the dichotomy between copiotrophs, which often encounter resources by motility, and oligotrophs, which are non-motile and rely on diffusive encounters [68,69]. A challenging evolutionary experiment to test the impact of the confluence on microbial diversity could include monitoring microbial diversity in two mesocosms, each seeded with identical initial microbial populations, but where one or more encounter mechanisms are suppressed in one of the mesocosms. For example, we expect that decreasing turbulence by suppressing mixing or eliminating motility using non-motile mutants in the starting population will decrease diversity in that mesocosm relative to the control. Ultimately, more work is needed to test the hypothesis that the confluence of encounter mechanisms can contribute to drive microbial diversity and the extent to which this may occur.

## 5. Microscale interactions are primed by encounters

We now describe several important examples of microbial interactions where quantifying encounter rates allows one to put lower bounds on the timescales of the interactions (figure 3). Throughout, we also illustrate the limitations of the simple kernels in equations (3.1)–(3.4).

Bacterial mortality is controlled by viral infections (figure 3*a*) and predation by protists (figure 3*b*) [17]. Owing to the small size of viruses, viral encounters with bacteria are diffusive in nature and can thus be modelled using equation (3.1) [38]. With the values *r*_bac_ = 1 μm, *r*_vir_ = 100 nm, *μ* = 1 mPa s and *T* = 293 K, one obtains from equation (3.1) the kernel $\Gamma =32.6\;\mu m^{3}\;s^{-1}$. At typical concentrations *c*_bac_ = 10^6^ ml^−1^ and *c*_vir_ = 10^7^ ml^−1^ of bacteria and viruses [36], respectively, this encounter kernel predicts that any given bacterium encounters one virus every $1/(\Gamma c_{\mathrm{vir}})\approx 50\;\min$, whereas any given virus encounters one bacterium every $1/(\Gamma c_{\mathrm{bac}})\approx 8.5\;h$. If the bacteria are motile, that further increases the diffusive encounter rate by a multiplicative dimensionless factor called the Sherwood number, which in the bacterium–virus case can increase the encounter rate up to twofold under typical conditions [38].

The far less abundant but larger protozoa (e.g. *c*_pro_ = 10^3^ ml^−1^ for heterotrophic flagellates [33,34]) may swim or create feeding currents through the beating of their flagella to graze on bacteria. Flows generated by beating flagella can enhance diffusive nutrient uptake, as described by the squirmer model [70,71], but, to the best of our knowledge, no formulae for encounter kernels exist to describe the capture of bacteria by such flows. Recent work has estimated the encounter kernel (also called clearance rate in the case of predation) from experimentally measured time-averaged flow fields for different flagellated species to be in the range $\Gamma \approx 10^{2}\text{–}10^{6}\;\mu m^{3}\;s^{-1}$ for organisms in the size range about 5−100 μm [18]. This estimate assumed non-motile bacteria (i.e. bacteria were modelled as tracer particles). As a result, taking $\Gamma =10^{4}\;\mu m^{3}\;s^{-1}$ shows that any given protist encounters one bacterium every $1/(\Gamma c_{\mathrm{bac}})\approx 2\;\min$, generally consistent with the observed ingestion rates of 2–20 bacteria per protist per hour [33]. Similarly, any given bacterium encounters one protist every $1/(\Gamma c_{\mathrm{pro}})\approx 1\;d$, which is consistent with the typical lifespan of a bacterium in the ocean [72]. The fact that a bacterium encounters a virus thirty times more frequently compared to encountering a protist should, however, not be taken to imply that mortality due to viral infection is more important than mortality due to protistan grazing, and is rather associated with the fact that viruses have typically a narrow host range [73] (making our calculation above an overestimate) whereas protists are often omnivorous. The two pathways of bacterial mortality are likely equally important [17].

Encounters between phytoplankton cells following a phytoplankton bloom (figure 3*c*) determine the formation of marine snow, which fuels the ‘biological pump’, the vertical export of carbon to the deep ocean that represents one of the climatically most important carbon fluxes in the ocean [2]. This coagulation process has been traditionally modelled as encounters between spherical cells sinking, often in the presence of turbulence, through equations (3.2) and (3.3), while neglecting diffusive encounters owing to the large size of phytoplankton cells [19,74]. On the other hand, phytoplankton come in a variety of shapes [75] with a majority of them being elongated [76]: to account for this, in recent work we have generalized equations (3.2) and (3.3) to the case of elongated cells [20,21,29], resulting in several new predictions regarding the timescales and nature of the bloom clearance dynamics. First, that work showed that identical buoyant elongated cells, owing to their orientation-dependent sinking/rising velocity, can encounter each other frequently even in the absence of turbulence [21], something that identical spherical cells cannot do because they all sink or rise at the same speed (as can be seen by setting *r*_A_ = *r*_B_ in equation (3.3), leading to *U*_A_(*r*_A_) = *U*_B_(*r*_B_)). Second, in a quiescent fluid a generalization of equation (3.3) to dissimilar elongated cells revealed that the formation of elongated aggregates is oscillatory in nature, with periodic bursts of several days in the concentration of aggregates of different sizes, whereas spherical aggregates reach a time-independent steady state [20]. Third, including the effect of turbulence in the case of identical elongated cells [29] revealed that elongation can increase encounter rates in turbulent flows by an order of magnitude as compared to spherical cells. Consequently, the formation of aggregates may be accelerated by a similar factor, providing a potential explanation for the rapid clearance of blooms of elongated or chain-forming phytoplankton species. Encounter kernels for dissimilar elongated cells in turbulence and for oblate cells in turbulence remain currently unknown.

Once marine snow particles have formed by coagulation, they sink and thereby export carbon to the deep ocean. The efficiency of this process is controlled by bacteria, which colonize and degrade the sinking particles: the rate of bacteria–particle encounters is here relevant for determining how rapidly particles get colonized by new bacteria (figure 3*d*) [7,10,77]. Consider a particle that has sunk below the mixing layer and is thus not subject to turbulent mixing. For non-motile and spherical bacteria (*U*_swim_ = 0), and in the typical case where the particle radius *r*_p_ is much larger than the radius of the bacterium *r*_bac_ (*r*_p_ ≫ *r*_bac_), one cannot apply the buoyant kernel in equation (3.3) directly, because sinking particles create a flow around them that can advect bacteria along the flow streamlines (shaded surfaces in figure 3*d*). When the diffusivity of bacteria is neglected (*D*_bac_ = 0), bacteria strictly follow the fluid streamlines of the flow created by the sinking particle and bacteria–particle encounters are then driven solely by ‘direct interception’, characterized by the kernel $\Gamma =1.45\pi r_{\mathrm{bac}}^{2}U_{\mathrm{sink}}$ [78], where *U*_sink_ is the sinking speed of the particle. Interestingly, this kernel depends only on *r*_bac_ and the sinking speed *U*_sink_ and does not depend on the particle size (as long as *r*_p_ ≫ *r*_bac_). When the diffusivity of bacteria is important (*D*_bac_ > 0), the encounter process can be described by a variant of equation (3.1) given by $\Gamma =4\pi D_{\mathrm{bac}}r_{p}Sh$, where the impact of the fluid flow induced by the sinking particle on the encounter process is encoded in the Sherwood number *Sh*, a dimensionless parameter that is a function of the Péclet number *Pe* = *r*_p_*U*_sink_/*D*_bac_ [22]. As an aside demonstrating the need for more work on encounter kernels, we mention that, for large Péclet numbers (*Pe* → ∞), it is not clear to us how the Sherwood-corrected diffusive kernel reduces to the interception kernel since the two predict different asymptotic limits. Note that the diffusive kernel also applies to encounters between viruses and swimming bacteria, as we mentioned above, but the Péclet number is moderate in this case (*Pe* ≈ 3).

For motile bacteria (*U*_swim_ > 0), the encounter process with particles depends sensitively on the interaction between bacterial swimming, bacterial shape and fluid flow induced by the particle. Motility can enhance effective bacterial diffusivity by up to three orders of magnitude as compared to non-motile bacteria, thus increasing the encounter rate by the same factor [7,77]. However, approximating the encounter process as diffusive is only valid as long the as the bacterial run length *λ* is much smaller than the particle radius (*λ* ≪ *r*_p_). Here, *λ* refers to the typical length of a segment of bacterial trajectory between subsequent tumble or reorientation events. For small sinking particles, which are also most abundant [30], this condition breaks down and the encounter process is then ballistic, rather than diffusive. One may then be tempted to apply equation (3.4) or its variants. However, the classical ballistic kernels assume straight-line trajectories, while the fluid shear induced by the sinking of the particle can bend the trajectories by reorienting motile bacteria as they approach the particle, and this bending strongly depends on the bacterial shape. As a result, shear and shape exert a strong control on encounters in the ballistic range [9]. Specifically, for elongated bacteria, shear can enhance encounters with slowly sinking particles (*U*_sink_ ≈ *U*_swim_) via redirecting bacteria to the leeward (downstream) side of the particle, but almost entirely prevents motile bacteria from attaching to fast sinking particles (*U*_sink_ ≫ *U*_swim_) via hydrodynamic ‘screening’ [9].

## 6. Tactic behaviour

So far, we focused on examples of encounters where behavioural responses of organisms were either not important or neglected; however, one of the big open challenges in the study of encounter rates is the incorporation of behavioural responses in the description of the encounter process [27]. Motile organisms often do not move randomly, but display tactic behaviours, i.e. they tune their motility in response to local environmental stimuli, which can be of chemical or hydrodynamic nature [7,79]. For example, chemical communication was found to be key for mate finding in copepod species [80]. Female individuals leave pheromone trails behind, which can be sensed and followed by male individuals. In this case, an approximate kernel reads $\Gamma =U_{m}^{⊥}\sigma$, where *σ* is the effective cross-sectional area of the trail, $\sigma \propto L\sqrt{LD_{p}/U_{f}}$ [81] (with *D*_*p*_ pheromone diffusivity, *U*_*f*_ female swimming speed and *L* trail length), and $U_{m}^{⊥}$ is the projection of the male swimming velocity perpendicular to the trail. Trail-tracking was observed to increase male–female encounters by factors of 20–80 compared to an unbiased random search [7,82].

Another well-known example of behaviour that impacts encounters is bacterial chemotaxis, the ability of bacteria to follow chemical gradients [83,84]. Using chemotaxis, cells are able to bias their motion towards regions where the concentration of attractant compounds is higher, thus increasing encounters with the sources of such compounds, such as leaking or lysed phytoplankton cells [26,85] or marine snow particles [86]. Extending encounter kernels through parameters that describe chemotactic behaviour—and behaviour in general—and how it interacts with the fluid flow is mostly an open challenge. Recent progress in modelling sperm chemotaxis towards eggs in marine invertebrates points towards general trade-offs between chemotactic behaviour and the distortion of concentration fields of chemoattractants into filaments [87].

## 7. Stochasticity and heterogeneity

Together with the important modifications linked to behavioural responses, the description of encounters in terms of equation (2.1) also finds limitations when considering a low numbers of objects, or heterogeneity in the distribution of objects, and needs to be adapted appropriately. The heterogeneity is particularly important in spatially structured environments, such as soil [88] or human gut [89].

Equation (2.1) is deterministic and applicable to well-mixed systems. As described earlier, an experimentalist counting all encounters between objects A and B during time *T* in an observation domain of volume *V* should obtain, on average, $\Gamma c_{A}c_{B}VT$ encounters. However, if the number of encounters that have been observed is small, stochastic effects must be taken into account [66,90]. Specifically, the deterministic description of equation (2.1) holds as long as

7.1 $$VT\gg \frac{1}{\Gamma c_{A}c_{B}},$$

that is, many encounters must occur in the ‘space–time volume’ *VT*. If this condition is violated, the observed number of encounters will fluctuate around the mean, with fluctuations of the order of $\sqrt{\Gamma c_{A}c_{B}VT}$ (see the chemical Langevin equation in [90] and algorithms therein to simulate stochastic encounters). Stochasticity can have major effects on cell interactions as illustrated by the stochastic ‘Kill the Winner’ model [66], whereby stochastic effects can lead to the total extinction of interacting predator–prey populations.

Spatial heterogeneity in the distribution of objects (chemicals, cells, particles) can create interaction hotspots [69]. Spatial heterogeneity can be accounted for in equation (2.1) by promoting concentrations to be functions of position: the encounter rate is then equal to $\Gamma c_{A}(x)c_{B}(x)$, where *x* is the spatial coordinate. Interaction hotspots arise due to the nonlinear dependence on cell concentrations. For example, constraining the same number of objects to occupy only half of an observation domain of volume *V* locally quadruples the encounter rate in the half-occupied domain, and, in the full domain, doubles the total encounter rate as compared to the uniform distribution. We refer the reader to Stundzia & Lumsden [91] for numerical methods to handle both stochastic and spatial effects.

## 8. Conclusion

In summary, encounter kernels are mathematical formulae that quantify the dependence of encounter rates between members of microbial ecosystems on cell phenotypes and biophysical properties, such as cell size, shape, density, diffusivity, motility patterns and environmental parameters, such as turbulence and fluid viscosity. Once the appropriate kernel together with cell densities are specified, equation (2.1) estimates encounter rates and thus the timescales at which interactions occur. While kernels have been studied for over a century, they were typically derived to model physical processes, such as collisions between molecules in gases, diffusive aggregation of colloids or rain formation. The applicability of physics-based kernels to faithfully represent the complexity of microbial interactions is thus limited, as exemplified by the impact of cell elongation on encounters, prompting further fundamental research on how cell phenotypes impact encounter rates. Such an encounter-centric approach should be particularly beneficial for the quantification of the rates of horizontal gene transfer, since all major horizontal gene transfer pathways are primed by encounters. Similarly, while often overlooked, kernels also provide concrete estimates of the nonlinear coupling coefficients in Lotka–Volterra type ecosystem models [6]. In these models, kernels can help to significantly constrain the otherwise vast space of the nonlinear interactions. Finally, kernels can inform the rational design of microbial interactions by providing guidance on how to suppress or enhance the interactions by eliminating or promoting encounters, providing a powerful link between experimental and modelling efforts [5].

## Data Availability

The relevant study data are displayed in the paper.
